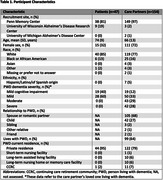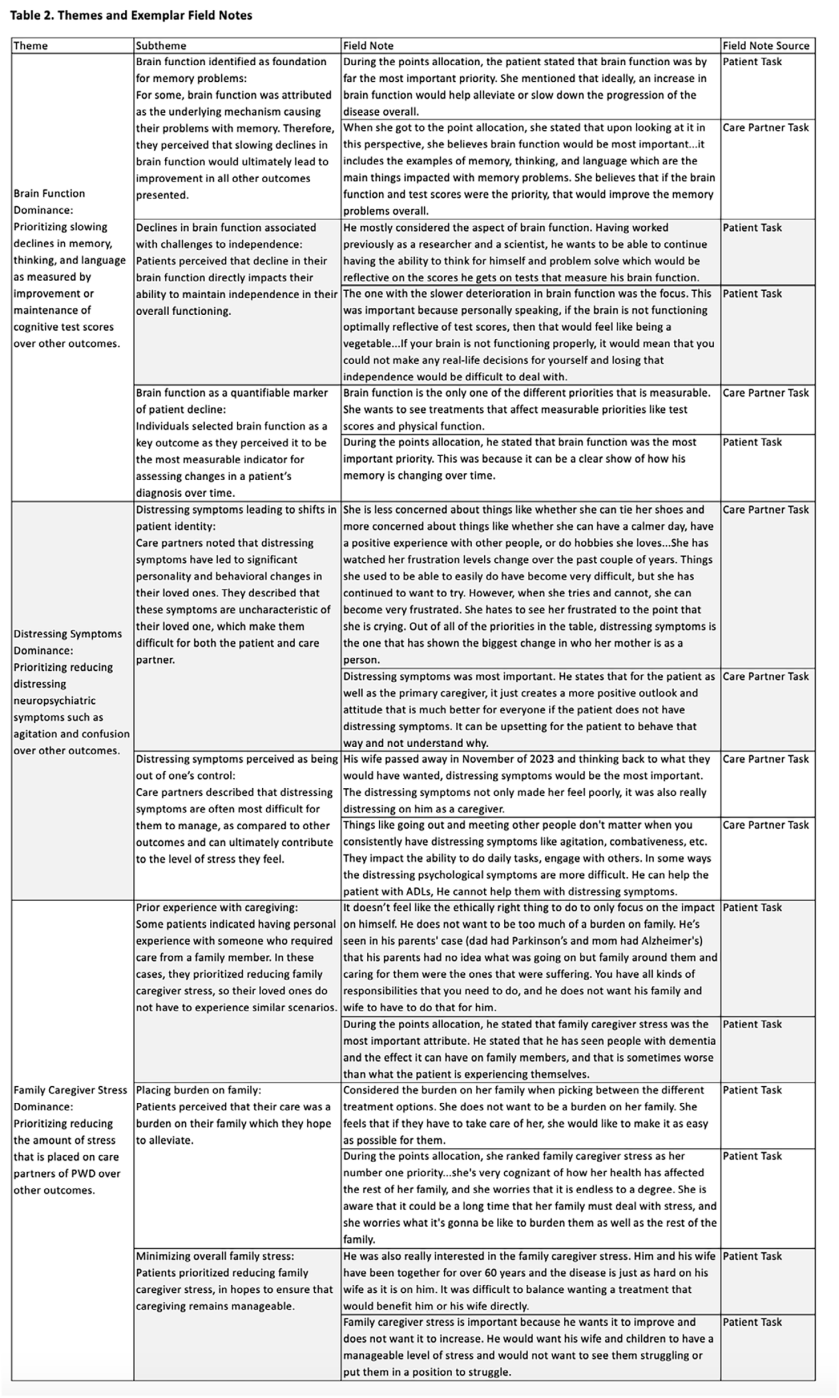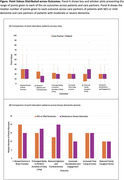# Evaluation of Patient and Care Partner Values when Considering Dementia Treatment Outcomes using a Point Allocation Task

**DOI:** 10.1002/alz70858_107383

**Published:** 2025-12-26

**Authors:** Maayra I Butt, Carolyn Chow, Paola Rosa, Josiah Drakes, Melanie Bahti, Casey Whitman, Annalise Rahman‐Filipiak, Allison M Randall, Lindsay R Clark, Raghuram Iyengar, Kristin Harkins, Jason Karlawish, Scott D Halpern, Catherine L Auriemma

**Affiliations:** ^1^ Palliative and Advanced Illness Research Center, Perelman School of Medicine at the University of Pennsylvania, Philadelphia, PA, USA; ^2^ Department of Psychology, University of Nevada, Las Vegas, Las Vegas, NV, USA; ^3^ Department of Medicine, Perelman School of Medicine at the University of Pennsylvania, Philadelphia, PA, USA; ^4^ Michigan Alzheimer's Disease Research Center, Ann Arbor, MI, USA; ^5^ Wisconsin Alzheimer's Disease Research Center, University of Wisconsin School of Medicine and Public Health, Madison, WI, USA; ^6^ The Wharton School, University of Pennsylvania, Philadelphia, PA, USA; ^7^ Penn Memory Center, Perelman School of Medicine at the University of Pennsylvania, Philadelphia, PA, USA; ^8^ Leonard Davis Institute of Health Economics, Perelman School of Medicine at the University of Pennsylvania, Philadelphia, PA, USA

## Abstract

**Background:**

Persons living with dementia (PWD) and their family care partners may prioritize possible treatment outcomes differently. We sought to understand how PWD and their family care partners value hypothetical treatment outcomes relative to one other using a point allocation task.

**Method:**

Participants distributed 100 points across six potential outcomes based on perceived relative importance. Outcomes included slowed decline in brain function, reduced agitation and combativeness, reduced family caregiver stress, prolonged independence in performing activities of daily living, increased time living at home, and increased socialization and engagement. Equality of distributions between outcome scores and between subgroups were compared using Wilcoxon signed‐rank and rank‐sum tests, respectively. To ensure comprehension and understanding of rationale for point distribution, we engaged participants in a “think out loud” approach as they completed the point allocation task. Qualitative data was documented in field notes and reviewed by the research team independently and during consensus meetings to identify relevant themes.

**Result:**

201 individuals participated (*n* = 154 [77%] care partners and *n* = 47 [23%] PWD; Table 1). Across all participants, slowing declines in brain function scored highest (20 [IQR 10, 30], *p* < .05 for all pair‐wise comparisons). Care partners compared to patients more highly valued reduced agitation and combativeness (15.5 [IQR 10, 25] vs 10 [IQR 3, 16)], *p* < .001). Patients compared to care partners more highly valued reducing family caregiver stress (16 [IQR 10, 30] vs 10 [IQR 5, 20], *p* = .003). Among care partners, those whose loved one had mild dementia more highly valued slowing declines in brain function (20 [IQR 10, 37.5] vs 15 [IQR 10, 25], *p* = .02). Conversely, care partners of those with moderate or severe dementia more highly valued reducing agitation and combativeness (20 [IQR 10, 25] vs 12.5 [IQR 7.5, 21] *p* = 0.007). Qualitative analysis revealed key themes regarding participant rationale for prioritizing certain outcomes over others (Table 2).

**Conclusion:**

PWD and dementia care partners expressed differing values when considering treatment outcomes. The development of future treatments should incorporate measurement of a range of valued outcomes critical to patients across the spectrum of impairment and their families.